# *Bidens meyeri* (Asteraceae, Coreopsideae): a new critically endangered species from Rapa, Austral Islands

**DOI:** 10.3897/phytokeys.42.8408

**Published:** 2014-10-13

**Authors:** Vicki A. Funk, Kenneth R. Wood

**Affiliations:** 1 US National Herbarium, Department of Botany, Smithsonian Institution, PO Box 37012, Washington, DC 20013-7012, USA; 2 National Tropical Botanical Garden, 3530 Papalina Road, Kalaheo, HI 96741, USA

**Keywords:** Asteraceae, Austral Islands, *Bidens*, Coreopsideae, Compositae, conservation, endemic, French Polynesia, IUCN Red List Category, Oceania, Rapa Iti.

## Abstract

*Bidens meyeri* (Asteraceae/Compositae) is described and illustrated from Rapa, Austral Islands, (French Polynesia). This new species is presumed to be most closely related to *Bidens saint-johniana* from nearby Marotiri Island. *Bidens meyeri* may be distinguished from *Bidens saint-johniana* based on the length of the peduncle (3 cm versus 10 cm), apex of the inner involucral bracts (glabrous vs. puberulent), smaller leaves (2.0–2.3 cm vs. 5–6 cm), and the general smaller size of the new species. Known from less than 50 individuals and restricted to one remote location, *Bidens meyeri* falls into the IUCN Critically Endangered (CR) category. The new species is named in honor of Dr. Jean-Yves Meyer, Délégation à la Recherche, Polynésie Française,

## Introduction

The Austral Islands are situated in the Southern Pacific and are part of French Polynesia. The Archipelago lies south of the Society Islands and consists of seven main islands of volcanic origin, and one atoll (Maria). The high islands include Rurutu, Tubuai, Rimatara, and Ra’ivavae as well as Rapa (27°36'00"S; 144°17'00"W), the second largest island (i.e., 40 km^2^). Rapa is about 5 million years old and it is very rugged, characterized by steep central ridges, mist shrouded spires, and towering black basalt sea-cliffs (Clarke 1971, Clouard and Bonneville 2005, Appelhans et al. 2014). The highest peak, Mont Perau (ca. 650 m), is covered by a small area of cloud forest (Meyer 2010).

Rapa is a high volcanic island and its climate is considered to be wet subtropical / subtemperate with a mean annual temperature of 20.6°C, a minimum at 8.5°C, and mean annual rainfall of 2500 mm at sea level (Barsczus 1980; Meyer 2011). Levels of endemism have been reported as very high among certain organisms, such as weevils (Paulay 1985). Area, altitude, and nearest land mass all have an effect on the evolution of the biota in these islands with Rapa being the second largest, highest, and most distant of all of the Austral islands. It is nearly 1200 km southeast of Tahiti, 3700 km northeast of the north island of New Zealand, and 8500 km southwest of the Baja Peninsula in Mexico. Threats to the biodiversity are primarily a result of burning, grazing (in particular goats and cattle), and invasive alien plant species.

Rapa has 238 native taxa of flowering plants and ferns including infraspecific categories, 85 of these are endemic to the Austral islands (35%), 73 of these are single island endemic taxa (30%) including this new species of *Bidens*. Considering only flowering plants, Rapa has 152 native flowering plant taxa, of these 65 (43%) are endemic to the Austral islands and 53 (35%) are endemic to Rapa (Meyer 2002, Wood 2002, Wood pers. com.). Two island endemic genera in the Compositae have been reported: *Apostates* N.S. Lander in the Madieae tribe, part of the Heliantheae Alliance, and *Pacifigeron* G.L. Nesom in the Astereae. Also, there are endemic Compositae species on Rapa that belong to two ‘endemic to Polynesia’ genera, namely *Fitchia* Hook. f. and *Oparanthus* Sherff both in the Coreopsideae tribe which is also part of the Heliantheae Alliance (Florence 1997, Shannon and Wagner 1997, Wagner and Lorence 2011).

**Figure 1.**
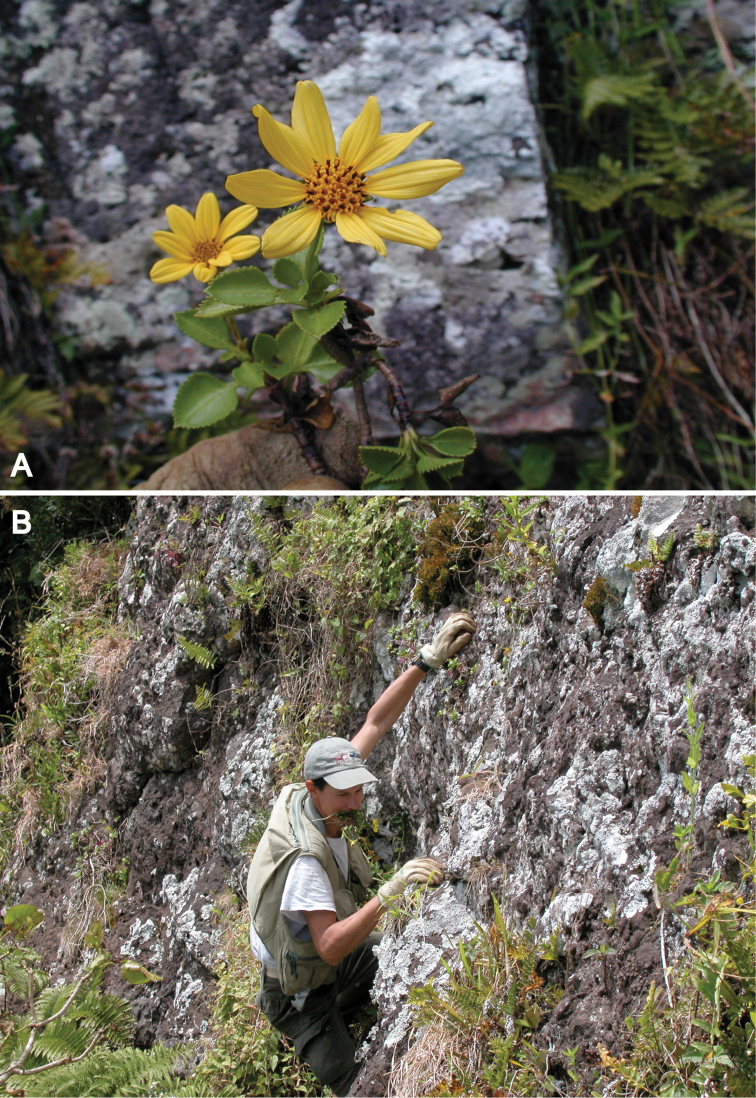
Photos of *Bidens meyeri*: **A** Close up of a flowering plant, note the gloved finger holding the plant **B** J-Y Meyer climbing with *Bidens* in his teeth, note yellow flowering plant on the cliff face just above his left hand. [Photo credits: **A** by J-Y Meyer; **B** by R Englund; both taken 16 Dec 2002].

In March – April 2002, during an expedition supported by the National Geographic Society, a group of scientists from the New York Botanical Garden (NYBG); the Délégation à la Recherche, Polynésie Française; and the National Tropical Botanical Garden (NTBG), Kaua`i, Hawai`i, conducted a botanical survey of the island of Rapa. They expected to stay there for a month. In fact, because of a logistic problem that delayed the supply ship (their means of transportation), several of them stayed for two months. During this Rapa expedition a number of very interesting taxa were discovered, one of which was a *Bidens* that could not be placed into any existing species (Fig. 1; Meyer 2002, Wood 2010).

**Figure 2.**
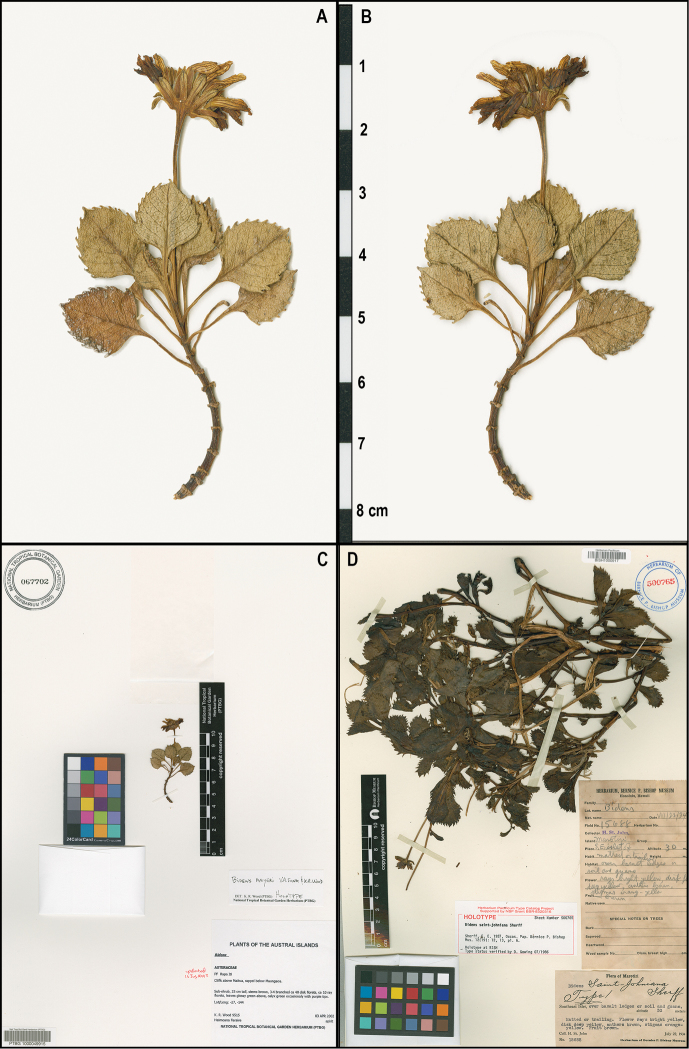
Photos of *Bidens* type specimens: **A–C** *Bidens meyeri* holotype (PTBG): **A–B** Specimen before mounting **A** Side with the involucral bracts and (mostly) upper surface of the leaves showing **B** Side with the flowers and (mostly) the undersurface of leaves showing **C** Holotype of *Bidens meyeri*, housed at PTBG **D** Holotype of *Bidens saint-johniana*, housed at BISH. [Photo credits: A–C by Jesse Adams, PTBG; D sent by BISH].

Describing this taxon was unusually difficult because of the scant material (Fig. 2a, b). Other samples were collected during the expedition but are inaccessible. The collector of the holotype specimen gathered several isotypes that would have been sufficient, however, all except the small one he retained (the type) have evidently been misplaced and with the untimely death of the expeditions team leader, Dr. Timothy Motley (NY followed by ODU), the specimens have not been available for study. On a subsequent expedition in December of 2002, an additional collection was made by Jean-Yves Meyer close to the original location (*Meyer 2315*; Fig. 1). Meyer’s collection was sent to the Paris herbarium (P) but cannot now be located (Meyer, pers. com.). After waiting for over ten years we have decided to go forward with the description of this new taxon as it is being included in a forthcoming molecular analysis and needs to be recognized for future conservation efforts. The leaf sample for the molecular analysis was taken from the holotype, prior to its designation as a holotype, with permission from the National Tropical Botanical Garden.

## Taxonomic treatment

### Bidens meyeri

Taxon classificationPlantaeAsteralesAsteraceae

V.A. Funk & K.R. Wood sp. nov.

urn:lsid:ipni.org:names:77142603-1

Fig. 2A–C

#### Type.

Austral Islands. Rapa, cliffs above Maitua, rappel below Maungaoa, 3 Apr 2002, *K.R. Wood & Heimoana Faraire 9515* (holotype: PTBG-067702!); 27°37'30"S; 144°20'20"W.

#### Description.

***Sub-shrub*** with 3–4 branches growing on cliff faces, ca. 25 cm tall, small side branches ca. 8 cm tall; *stems* brown, glabrous, lower portion smooth, upper portion striated, ca. 1 mm wide (when dry), glabrous, older nodes with the remains of leaf bases. ***Leaves*** opposite, simple, somewhat fleshy, glabrous, without true petioles but looking petiolate because of narrowed blade bases, 2.1–2.3 cm long; *broad part of leaf* ovate, glossy green above, dull green below, 1.1–1.5 cm long × 1.0–1.2 cm wide; *margins* of broad part of leaf dentate with teeth curved toward apex and with mucronate tips, 6–9 teeth per side; *apex* mucronate or apiculate; *venation* pinnate usually with one lateral vein for each tooth; *narrow portion of leaf* ca. 1 cm long decurrent with leaf bases wrapping around stem and nearly touching one another. ***Heads*** solitary, ~ 1 cm in diameter (excluding rays), peduncle 3 cm long, glabrous with prominent ribs (when dried); *involucral bracts* in 2–3 rows, outer two rows bright green, purple tipped in some, slightly fleshy, glabrous with 3 prominent veins (when dried), arched outward, ca. 5 mm × 1.3 mm (at the broadest point near the apex), apex rounded with a small acute tip; innermost row (may also be outer row of receptacular bracts) lanceolate, brownish with lighter hyaline margins, glabrous, 5.5 mm × 1.1 mm (at widest point near the base). ***Ray flowers*** yellow with many veins, 8–9 per head, sterile, ca. 9 mm long (including 2 mm tube) × 1.5–2.0 mm wide; *disk flowers* ca. 30–40, perfect; *corollas* yellow, glabrous; *anther* thecae dark, pollen yellow; *style* branches yellow. ***Pappus*** of 2 very short irregular awns with scattered hairs but without barbs. ***Achenes*** immature but apparently dark colored and glabrous, at least near the apex.

#### Additional collection.

Austral Islands: Rapa, Tevaitau on cliff, only two plants seen, 272 m, on bare rock, 16 Dec 2002, *J.-Y. Meyer 2315* (Specimen hopefully at P). Fig. 1.

#### Distribution and ecology.

The type of *Bidens meyeri* was discovered during a rappel utilizing ropes and climbing-saddle around the windswept mesic cliffs above Maitua, Rapa, French Polynesia. The holotype location is the only population of any size; the second collecting site had only two individuals. The type locality can be described as a windswept mesic cliff habitat with small ledges and pockets of granular soil, bordered by steep slopes interspersed with herbs and low-statured native forest and shrubland. The aspect is northeast with a 70% open canopy for exposure to sun. Associated tree species include *Oparanthus coriaceus* (F. Br.) Sherff, *Oparanthus rapensis* (F. Br.) Sherff, *Corokia collenettei* Riley, *Fitchia rapense* F. Br., Metrosideros collina (J. R. Forst. & G. Forst.) A. Gray var. villosa (L.) A. Gray, *Apetahia margaretae* (F. Br.) Wimmer, and *Sophora rapaensis* H. St. John. Shrubs, vines, and herbs include *Plantago rupicola* Pilg., *Dianella intermedia* Endl. var. *punctata* F. Br., *Veronica rapensis* F. Br., *Kadua rapensis* F. Br., *Alyxia stellata* (J.R. Forst. & G. Forst.) Roem. & Schult., *Dichelachne crinita* (L. f.) Hook. f., and some *Freycinetia arborea* Gaudich. Associated ferns include *Blechnum attenuatum* (Sw.) Mett., Blechnum vulcanicum (Blume) Kuhn var. rapense E.D. Br., *Sphaeropteris medullaris* (G. Forst.) Bernh., *Alsophila stokesii* (E.D. Br.) R.M. Tryon, *Polystichum rapense* E.D. Br., *Belvisia dura* (Copel.) Copel., *Thelypteris margaretae* (E.D. Br.) Ching, *Davallia solida* (G. Forst.) Sw., *Selaginella arbuscula* (Kaulf.) Spring, *Nephrolepis exaltata* (L.) Schott, and *Pteris comans* G. Forst (Wood 2002, pers. com.). *Meyer* 2315 was collected at a site with *Pyrrosia serpens* (G. Forst.) Ching, *Peperomia* sp., *Verbena litoralis* Kunth, *Commelina diffusa* Burm. f, *Davallia solida* (G. Forst.) Sw. and *Psilotum nudum* (L.) P. Beauv (pers. com.).

#### Etymology.

The new species is named in honor of Dr. Jean-Yves Meyer, friend and conservation biologist at the Délégation à la Recherche, Polynésie Française, in recognition of his research of this species and his efforts in exploring and conserving the unique biota of Rapa (e.g., Meyer 2011).

## Discussion

The only other native species of *Bidens* from the Austral Islands is *Bidens saint-johniana* Sherff (1937) found on Marotiri, a group of small rocky islets located ca. 80 km southeast of Rapa. Marotiri has been surveyed only twice by botanists: St. John and Forges. *Bidens saint-johniana* was first collected at the Southeast Islet, 22 July 1934, by Harold St. John (Fosberg 1972; St. John 1982) and his assistant at the time, Ray Fosberg (*St. John 15683*; holotype: BISH; isotype F, http://plants.jstor.org/specimen/f0075334f?history=true; images of both were examined; Fig. 2D), and again in 1979 by B. Richer de Forges (*Nicolas Hallé 6860*, P; Hallé 1980). *Bidens saint-johniana* is a much more robust plant than *Bidens meyeri*, its leaves are larger (total length 5–6 cm, width at the widest part 3.5–4.0 cm) and peduncle longer (10 cm vs. 3 cm), and the apex of the inner involucral bracts is puberulent (vs. glabrous). Based on the images of the holotype (BISH; Fig. 2D) and isotype (F) the leaves are not thickened and the teeth are larger and not as curved and do not have a mucronate tip. Finally the length of the side branches was 14–15 cm as opposed to those of *Bidens meyeri* which are less than 8 cm.

When the new species was run through the key in the *Bidens* treatment written by Welsh (1998) covering the Society Islands, it did not key out to anything remotely similar and it did not fit any of the descriptions. It does, however, have some superficial resemblance to *Bidens molokaiensis* Sherff and *Bidens mauiensis* Sherff from Hawaii, as Sherff (1937) observed.

Rapa’s flora is usually mentioned as being closely allied to that of New Zealand and Australia. However, after evaluating the results of numerous exceptions, some botanists (e.g., van Balgooy 1971) consider Rapa to be an ‘anomalous district’ in the SE Polynesian Province, and the high levels of endemic biological diversity in both the flora and fauna still puzzle many scientists because of the island’s relatively small size. There are 53 flowering plant species (35%) that are single island endemics to Rapa, including three endemic plant genera, namely *Apostates* (Asteraceae: Bahieae), *Pacifigeron* (Asteraceae: Astereae) and *Metatrophis* F. Br. (Urticaceae). The new species is clearly related to the Pacific *Bidens* radiation (Hawaii & French Polynesia) rather than taxa found on Rapa’s neighbors to the South.

## Conservation status

Utilizing the World Conservation Union (IUCN) criteria for endangerment (IUCN 2001), we find that *Bidens meyeri* easily falls into the Critically Endangered (CR) category, and faces a very high risk of extinction in the wild. The IUCN alphanumeric summary of our evaluation of criteria and subcriteria is: B1ab(v); B2a, B2b(i–iii); D. These criteria are defined as: B1, extent of occurrence less than 100 km^2^; B1a, known to exist at only a single location; B1b(v) continuing decline inferred in number of mature individuals; B2, total area of occupancy less than 10 km^2^; B2a, one population known; B2b(i–iii), habitat continuing decline inferred; D, population estimated to number fewer than 50 individuals. Threats to *Bidens meyeri* include possible fires, habitat degradation and destruction by feral goats (*Capra hircus* L.), along with competition with non-native plant taxa especially *Psidium cattleianum* Sabine and, of course, climate change.

## Supplementary Material

XML Treatment for Bidens meyeri
